# Inconsistent findings for the eyes closed effect in children: the implications for interviewing child witnesses

**DOI:** 10.3389/fpsyg.2014.00448

**Published:** 2014-05-20

**Authors:** Marilena Kyriakidou, Mark Blades, Dan Carroll

**Affiliations:** ^1^Department of Law and CriminologyIntercollege, Larnaca, Cyprus; ^2^Department of Psychology, University of SheffieldSheffield, UK

**Keywords:** child eyewitness, eyes closed effect, police interviews, eyewitness testimony, child interviews, children's testimony

## Abstract

A child who alleges that they have been the victim of a crime will be interviewed by police officers. During a police interview it is important that the interviewer obtains the most accurate testimony possible from the child. Previous studies have shown that if children have their eyes closed during an interview they sometimes report more correct information. This paper includes two studies. In Experiment 1 156 children experienced an event and were then questioned about it. Half the children answered with their eyes open and half with their eyes closed. The participants with eyes closed provided more correct information. In Experiment 2 152 children answered questions in different conditions including eyes open and eyes closed conditions. In contrast to Experiment 1 there was no beneficial effect for the eyes closed condition. These inconsistent results are discussed with reference to actual police interviews. It is suggested that until there has been more research into eyes closed procedures caution should be taken in recommending such procedures for police interviews with children.

## Introduction

Children may be interviewed in many contexts and a particularly important one is when they are interviewed as part of a forensic investigation. Children may be interviewed because they have been witnesses to a crime or because they have been the victim of a crime (Ministry of Justice, 2011). In cases of physical or sexual abuse a child may be the only witness, because the nature of the crime means that evidence from other witnesses is unlikely, and in many cases there may be a lack of other evidence, such as medical signs (Jong, 1996). For example, Kyriakidou (2012) examined every case of child maltreatment in the Republic of Cyprus for a 5-year period (2004–2009) and found that in two-thirds of these cases the only source of evidence was the child's testimony itself. Therefore a child's testimony can be crucial for investigating an alleged crime and it is important that police interviewers obtain the most complete and accurate responses from a child witness.

Several questioning techniques have been developed for forensic interviewing. These include the Cognitive Interview (Fisher and Geiselman, 1992); Achieving Best Evidence (ABE) (Ministry of Justice, 2011); the P.E.A.C.E. (Preparing and planning, Engage and explain, Account, Closure and Evaluate) guidelines (Clarke and Milne, 2001), and the National Institute of Child Health and Human Development (NICHD) protocol (Lamb et al., 2011). Procedures like the NICHD protocol have been extensively investigated and have been shown to improve the quality of interviews with children (Lamb et al., in press). Nonetheless, the procedures may not always be fully implemented by interviewers (Hershkowitz et al., 2005; Westcott and Kynan, 2006) and there is still a need to consider interview techniques that can be used easily with children. This has led to research into the effectiveness of eyes closed procedures.

Adult eyewitnesses provide more accurate information, without an increase in incorrect information, when answering questions with their eyes closed (Wagstaff et al., 2004; Perfect et al., 2008; Vredeveldt et al., 2012; Vredeveldt and Penrod, 2012). In a series of five experiments with adults, Perfect et al. (2008) investigated the effects of an eye closure condition for recall of different types of presentation (video or live), question modality (visual or auditory) and question type (free recall or specific). In three of the experiments participants were shown different video clips and in the other two experiments participants took part in a live event. The participants were then interviewed while keeping their eyes open or closed. Overall Perfect et al. found that participants recalled more accurate information in conditions when they had their eyes closed, and this was particularly true for the recall of visual information. Similar effects for improved accuracy were found by Perfect et al. (2011), who replicated the improved accuracy of recall with eye closure, and also found that eye closure during recall tended to reduce the negative effects of auditory noise. The results of these studies suggest that eye closure is an effective procedure for increasing the accurate information provided by adult witnesses.

Similar positive findings have been found in studies with children. Mastroberardino et al. (2012) interviewed 6- and 11-year-olds in both eyes open and eyes closed conditions immediately after the children had watched a video clip from the film “Jurassic Park”. Children were asked for free recall and also asked specific questions. Children with their eyes closed recalled more accurate details without an increase of inaccurate details when they were asked specific questions, though there was no effect of eye closure on free recall. Natali et al. (2012) showed 11-year-olds a bank robbery from the film “Dog Day Afternoon” and tested the children's recall for the first time immediately after seeing the film, and then tested their recall a second time after a delay of a week. Children who had their eyes closed provided more correct information in free recall, and answered specific questions more accurately both immediately and after the delay.

Children and adults may be better at answering questions with their eyes closed than with their eyes open for several reasons. When interviewees have their eyes open the interview task has a dual aspect. The interviewee needs to generate answers to the questions at the same time as monitoring the environment. An interviewee has to pay attention to the interviewer and to any other people in the room, as well as taking into account any distractions, like noise, in the environment. According to the cognitive load hypothesis people have a limited amount of cognitive resources in any task, and the effort of monitoring the environment may interfere with the effort of retrieving information and reduce the quantity or accuracy of the responses (Glenberg, 1997). Closing eyes has the effect of removing or reducing the stimuli from the environment that a person experiences, thereby changing the nature of the task from a dual to a single task so that an interviewee can concentrate their cognitive resources on just the retrieval of relevant information (Glenberg et al., 1998; Perfect et al., 2008). Adults perform better in tasks when the distracting effect of the environment is lessened, for example, Glenberg et al. found that adults' recall was better when they were asked to look at a static image rather than a more complex moving image, and Perfect et al. (2012) found that increased distraction led to a reduction in adults' recall accuracy.

Children may be particularly affected by environmental cues in an interview context, because an interview involves face-to-face interaction with an adult. This interaction involves a cognitive load as the child has to process information from the interviewer's face, and the child also has to take into account any social cues implicit in another person's gaze (Doherty-Sneddon et al., 2001; Doherty-Sneddon and Phelps, 2005). Children may look away from an interviewer while they consider their answer, especially when responding to more difficult questions (Doherty-Sneddon et al., 2002, 2007; Doherty-Sneddon, 2004), and looking away is one way that children can disengage from the environment if that environment is distracting them from focusing on the retrieval of appropriate information (Doherty-Sneddon and Phelps, 2005). Asking children to close their eyes may have a similar effect by getting children to disengage from the environment during an interview.

Eye closure might also benefit recall if closing eyes results in better visual imagery (Ganis et al., 2004), and if in turn better visual imagery helps children to retrieve more visual information about a past event (Vredeveldt et al., 2011). If this is the case answering questions with eyes closed should particularly benefit the recall of visual details, but might not necessarily improve the recall of other (e.g., auditory) details, in which case eye closure would be modality specific.

If children give more information, and more accurate information when answering questions with their eyes closed, this has implications for interviewing children in forensic contexts. Asking children to keep their eyes closed seems to be a procedure that can be implemented easily. The procedure does not require interviewer training, or any alteration to the existing police guidelines for interviewing witnesses (Ministry of Justice, 2011) other than asking children to close their eyes.

As noted above there have only been a couple of published studies with children (Mastroberardino et al., 2012; Natali et al., 2012) both of which found positive effects when children answered specific questions about a film extract with their eyes closed. We carried out a similar study (Experiment 1, below), but unlike the previous studies that have examined children's recall of films we used an event that combined live and video elements so that children saw an actual event that was acted out in front of them. We used such an event to approximate more closely to a real life eyewitness experience. The children were interviewed either soon after the event or a week later.

Given the previous positive results for eye closure (Mastroberardino et al., 2012; Natali et al., 2012) we expected children would recall more information when interviewed with eyes closed than with eyes open immediately after the event. Following previous findings for both adults and children that eyes closed interviews are still beneficial after a delay (Natali et al., 2012; Vredeveldt et al., 2013) we expected children to recall more when interviewed with eyes closed than with eyes open a week after the event.

## Experiment 1

### Methods

#### Participants

Experiment 1 included 156 children aged 6–12 years, mean age 9 years (78 girls and 78 boys) from schools in Cyprus. The children were a random sample of the children available in schools at the time of testing. The children experienced an event and were then questioned about it. Children saw the event in groups of between 10 and 20 participants. Seventy-eight children interviewed with their eyes closed and 78 with their eyes open. In the immediate condition 79 children were interviewed within an hour of seeing the event. In the delay condition, 77 children were interviewed 7 days after the event.

Ethical approval was given by the Department of Psychology, University of Sheffield. Permission to work in schools was provided by the Ministry of Education in Cyprus and the parents of children gave informed consent for their children to take part.

#### Materials

The 10 min event was a combination of a scripted live performance (6 min) and a video (4 min) performed by three assistants (who took no further part in the study). In the live performance an assistant called “Kim” took participants to a room where she started to show them how to do a magic trick. While Kim was demonstrating the trick, her friend “Mik” ran into the room and said that she was upset because she had lost her favorite jacket and that she might have left it at Kim's house the evening before. Mik then described the jacket. Kim calmed Mik down and said that she had a video from the previous evening that might show what had happened to the jacket. Participants were asked if they minded watching the video. The video showed Kim and Mik at a table in a dining room where they were eating pizza and talking. Mik's jacket was shown hanging on a peg. Kim and Mik left the room to get ice cream, and while they were away, a female entered the dining room and took the jacket. When Kim and Mik returned they continued with their meal without noticing that the jacket had gone. Having seen the video Mik was shocked and said that she would go to the police. She then left the room. Kim apologized for the interruption and then finished the demonstration of the magic trick.

#### Procedure

After the event participants were questioned individually. The interviewer had not been present at the event. The study was carried out in Greek and therefore quotes are translations from the original. In the immediate condition the interviewer started by saying, “I would like to ask you some questions about the event you saw today.” In the delay condition the interviewer said, “I would like to ask you some questions about the event you saw last week.” In the eyes open condition children received no instructions about where they should look during the interview. In the eyes closed condition the interviewer added, “During the questioning I would like you to keep your eyes closed.” If children opened their eyes during the interview the interviewer reminded them to keep their eyes closed. In both conditions children were told, “If you don't know the answer to a question, it's okay, you can say that you don't know the answer.” The interviews were audio taped for later analysis.

Twenty-eight questions were used in the interview (see Appendix 1 in Supplementary material). Question 1 was a free recall question asking participants to freely recall the event and question 2 was an open-ended question that asked for a description of the person who stole the jacket. The intention was to analyse these questions separately, but several children included information about the person who stole the jacket when answering question 1, and therefore children's responses to both questions were combined and will be referred to as the free recall. The rest of the questions were specific ones that required children to generate an answer. There were no yes/no or forced choice questions. Twelve of the specific questions were about visual information (e.g., “What color was Kim's t-shirt when she was showing you the magic trick?”). Visual questions are numbered 5–16 in the Supplementary material. Fourteen of the specific questions were about auditory information (e.g., “What was the name of the girl who demonstrated the magic trick to you?”). Auditory questions are questions numbered 3–4 and 17–28 in the Supplementary material. The experiment was designed with equal numbers of visual and auditory questions, but two visual questions had to be dropped from the analysis. One visual question asked the children where they went to be interviewed and the other asked who took the child to the interview, but for practical reasons children were questioned in different places in schools and were taken there by different people. Children's ability to answer these questions partly depended on how familiar they were with the specific interview place or with the person taking them, and therefore the answers could not be coded consistently.

Questions 1–4 were asked first and in the same order for all participants. The rest of the questions, 5–28, were asked in a different random order for each participant. When the interview was completed the child was thanked and was asked not to talk about the interview with other children. We could not check whether children discussed the event or the interview with other children, but even if they did so there was no reason to believe that the number of children who discussed the event in the eyes closed condition would be more (or less) than the children in the eyes open condition.

#### Coding

As noted above the responses to questions 1 and 2 were combined and scored for the number of items of information provided, number of correct details, number of incorrect details, and confabulations. For information in a response to be considered as a relevant detail (correct or incorrect) it had to provide details about time, people (e.g., names, gender), objects (e.g., food, clothes), places (e.g., house, living room) or actions (e.g., “took the jacket”). These details were chosen because an analysis of actual police interviews (Kyriakidou, 2012) had shown that such details were frequently requested by interviewers. For example, one child, in free recall, said, “Give me a moment to remember. I remember something now. I remember two girls they were in a house and they ordered pizza. They were talking about different stuff until they left the house and another girl came in the house and took the jacket.” In this extract the child mentioned 8 details (underlined). Of these details, one, “they left the house” was incorrect; the others were correct. There were almost no confabulations, and therefore confabulations were ignored.

Answers to specific questions were scored as correct if a participant gave an appropriate response. Appropriate responses to specific questions were defined prior to the interview. If an answer to a specific question could include two potential details, children had to mention at least one detail in their answer to be correct, and for answers that could include three or more details children had to give at least two details. Children's answers were only scored as correct if they met these criteria. All other responses to specific questions (including wrong answers, and “don't knows”) were coded as incorrect, because such answers provide no forensic evidence. Two coders coded correct items in free recall and correct responses to specific questions of the interviews. Cohen's kappa was run to determine the level of agreement. There was good agreement for free recall, *k* = 0.383 (*p* < 0.001) and for specific questions *k* = 0.394 (*p* < 0.001).

### Results

Table 1 summarizes how the interview conditions (eyes closed or eyes open) and delay conditions (immediate interview or interview after 1 week) influenced children's recall in free recall and for the specific questions.

**Table 1:** **Mean scores for interview condition and for delay in Experiment 1**.

	**Free recall *M* (*SD*)**			**Specific questions *M* (*SD*)**		
	**No. of details**	**Correct details**	**Incorrect details**	**Correct answers**	**Correct visual**	**Correct auditory**
Eyes closed	8.9 (4.9)	7.8 (4.6)	1 (1.3)	11.1 (3.6)	6.2 (1.8)	4.9 (2.4)
Eyes open	7.2 (5.5)	5.9 (4.7)	1.7 (3.9)	10 (3.5)	5.7 (1.8)	4.3 (2.2)
Immediate	8.9 (5.8)	7.9 (5.2)	1.4 (3.9)	11.7 (3.8)	6.3 (1.8)	5.5 (2.5)
Week delay	7.2 (4.5)	5.9 (4)	1.3 (1.4)	9.4 (3)	5.7 (1.8)	3.7 (1.6)

#### Free recall

***Number of details***. To investigate the effects of interview and delay conditions on the number of details given in free recall a 2 interview (eyes closed, eyes open) × 2 delay (immediate, 1 week) ANOVA was carried out. The interview influenced the number of details provided to questions 1 and 2 [*F*_(1, 147)_ = 4.39, *p* = 0.038, η^2^ = 0.029]. Children provided more details with their eyes closed (*M* = 8.9, *SD* = 4.9) than with their eyes open (*M* = 7.2, *SD* = 5.5). There was an effect of delay [*F*_(1, 147)_ = 4.13, *p* = 0.044, η^2^ = 0.66]. Children provided more details in the immediate condition (*M* = 8.9, *SD* = 5.8) than after a week (*M* = 7.2, *SD* = 4.5), but there was no interaction between interview and delay.

***Number of correct details***. A 2 interview (eyes closed, eyes open) × 2 delay (immediate, 1 week). ANOVA was conducted on the number of correct details, there was an interview effect [*F*_(1, 147)_ = 6.97, *p* = 0.009, η^2^ = 0.05]. Children provided more correct details with their eyes closed (*M* = 7.8, *SD* = 4.6) than open (*M* = 5.9, *SD* = 4.7). There was an effect of delay [*F*_(1, 147)_ = 7.35, *p* = 0.008, η^2^ = 0.48] with children in the immediate condition recalling more correct details (*M* = 7.9, *SD* = 5.2) than children in the delay condition (*M* = 5.9, *SD* = 4). There was no interaction.

***Number of incorrect details***. There was no difference in the number of incorrect details provided in the interview conditions [*F*_(1, 147)_ = 2.22, *p* = 0.139, η^2^ = 0.02]. There was no effect of delay and no interaction between interview and delay on the number of incorrect details.

#### Specific questions

There were 26 specific questions therefore the maximum possible correct score for each child was 26. A 2 interview (eyes closed, eyes open) × 2 delay (immediate, 1 week) ANOVA was carried out. There were more correct responses [*F*_(1, 152)_ = 4.09, *p* = 0.045, η^2^ = 0.03] with eyes closed (*M* = 11.1, *SD* = 3.6) than with eyes open (*M* = 10, *SD* = 3.5). There was also an effect for delay [*F*_(1, 152)_ = 18.81, *p* < 0.001, η^2^ = 0.11] with more correct answers in the immediate condition (*M* = 11.7, *SD* = 3.8) than after a week (*M* = 9.4, *SD* = 3). There was no interaction.

***Visual questions***. The maximum possible score for correct answers to visual questions was 12. A 2 interview (eyes closed, eyes open) × 2 delay (immediate, 1 week) ANOVA was conducted on the visual questions. Children provided more correct answers to visual questions with their eyes closed (*M* = 6.2, *SD* = 1.8) than with their eyes open (*M* = 5.7, *SD* = 1.8) [*F*_(1, 152)_ = 0.04, *p* = 0.047, η^2^ = 0.03]. There was no effect of delay [*F*_(1, 152)_ = 3.66, *p* = 0.058, η^2^ = 0.03] and there was no interaction.

***Auditory questions***. The maximum possible number of correct answers for auditory questions was 14. A 2 interview (eyes closed, eyes open) × 2 delay (immediate, 1 week) ANOVA was carried out. Children's recall on the auditory questions was not influenced by the interview [*F*_(1, 152)_ = 2.2, *p* = 0.14, η^2^ = 0.03]. Delay did have an effect on the accuracy of children's auditory answers [*F*_(1, 152)_ = 27.26, *p* < 0.001, η^2^ = 0.15] as children gave more correct answers when questioned immediately after the event (*M* = 5.5, *SD* = 2.5) than when questioned a week later (*M* = 3.7, *SD* = 1.6).

### Discussion

In Experiment 1 children gave more details and more correct details about the event in free recall when they had their eyes closed, both immediately after the event and after a delay of a week. Keeping eyes closed had no effect on the number of incorrect details reported. For specific questions children answered more visual questions correctly when they had their eyes closed, both immediately and after the delay, and although the increase in the number of specific visual questions answered correctly was small in this experiment, the same effect could be important in an actual interview when children are asked much larger numbers of questions (see General discussion). There was no effect of eye closure when children answered auditory questions.

These results partially support previous research with children. Children's better performance with eyes closed in free recall was similar to Natali et al. (2012) who also found that children who had their eyes closed were more accurate when asked for free recall of a film they had seen, both immediately after seeing the film and a week later. However when Mastroberardino et al. (2012) tested children's free recall of a film immediately after viewing it they did not find better performance from children with their eyes closed. Adults generally perform better in free recall in eyes closed conditions (Perfect et al., 2008, Experiments 3 and 5; Vredeveldt and Penrod, 2012), but as yet the limited evidence from studies with children does not show a consistent benefit of eye closure in free recall. It is difficult to reconcile the results from Experiment 1 and Natali et al. with those of Mastroberardino et al. because all three studies used similar procedures, and if eyes closed has a beneficial effect it should be apparent in all cases of free recall.

Mastroberardino et al. (2012) found that children with eyes closed performed better than children with their eyes open when answering cued recall questions. The questions in Mastroberardino et al. asked children to provide additional details about the information they had already included in their free recall and could therefore have been a mix of questions about visual or auditory details. In Experiment 1 we distinguished between specific questions about the visual and auditory information in the event. Although we found that children in the eyes closed condition gave more correct responses to visual questions there was not a similar effect for auditory questions. This could suggest that for children eye closure is only effective in contributing to the recall of visual information and that eye closure is modality specific (Vredeveldt et al., 2011). But this suggestion cannot be maintained in the light of Natali et al.'s (2012) study because they found that eyes closed improved children's accuracy when answering both visual and auditory questions.

When children do perform better with their eyes closed it could be because eye closure allows children to focus on the task of answering questions by reducing distracting information from the environment (Perfect et al., 2008). In particular, a child with their eyes closed can avoid any distracting social cues that may be given by the interviewer (Doherty-Sneddon et al., 2001; Doherty-Sneddon and Phelps, 2005). However, in the course of Experiment 1 we noted that children in the eyes closed condition had difficulty keeping their eyes closed. Remembering to keep their eyes closed requires effort that could have distracted the children from answering the questions, and when an interviewer has to remind a child to close their eyes this interrupts the interview and the child's focus on the questions. Greater distraction may reduce recall (Glenberg et al., 1998; Doherty-Sneddon and Phelps, 2005; Perfect et al., 2012) and have a negative effect on children's performance. Therefore in Experiment 2 we considered whether children's recall was enhanced if they only closed their eyes at particular times during an interview. In this way children might still benefit from the positive effects of eye closure without being distracted by the effort of keeping their eyes closed continuously.

In Experiment 2 there were 4 interview conditions. In the first condition children were not given any instructions about closing their eyes and kept their eyes open throughout the interview. Therefore children had their eyes open during questioning and during answering and this will be referred to as the EO/EO condition.

Children in a second condition were asked to keep their eyes closed throughout the whole of the interview during both questioning and answering (EC/EC). Children in the EC/EC condition were expected to perform better than children in the EO/EO condition, in line with the results from Experiment 1.

In a third condition (EO/EC) children kept their eyes closed only while answering a question. In this condition children could benefit from seeing non-verbal cues from the interviewer that might increase the child's understanding of the question (Doherty-Sneddon and Kent, 1996), but were not distracted by the interviewer or external factors when they answered with their eyes closed (Doherty-Sneddon et al., 2001; Phelps et al., 2006) so we expected children would perform better when answering specific questions in the EO/EC condition than in the EO/EO condition.

Children in the fourth condition (EC/EO) closed their eyes only while listening to a question. Children in this condition were expected to perform no better than those in the EO/EO condition, because in the EC/EO condition they did not have the advantage of seeing the interviewer during the questioning, but did have the disadvantage of seeing the interviewer when answering.

## Experiment 2

### Methods

#### Participants

The study took place in Cyprus. There were 152 children (88 girls and 64 boys) aged between 9 and 13 years with a mean age 10.6 years (*SD* = 0.98). There were 39 children in the EO/EO condition, 39 in the EC/EC condition, 37 in the EO/EC condition, and 37 in the EC/EO condition. There was a similar age range and approximately equal numbers of girls and boys in each condition. Ethical permission was obtained from the Psychology Department of the University of Sheffield. Permission to interview the children was obtained from the Ministry of Education in Cyprus, the principals of each primary school and from the parents or guardians of each child.

#### Materials

Children were shown a video, in Greek, lasting 5 min 50 s called “Pet Shop” by Michael Gabriel Zenelis. The film begins by showing the owner of a pet shop taking care of the animals in the shop. The owner notices that one of the dogs has a problem with its leg. The dog is put on one side and the owner makes a telephone call asking his colleague to come to fetch the dog because it is disabled and unsuitable for selling. Meanwhile, in a nearby park, a group of boys are playing basketball. When the ball goes out of the playground it rolls in front of the main character of the film. This boy is counting his money and he refuses to join in with the other children when they ask him to play. The boy goes into the pet shop and wants to buy a particular dog, which is too expensive for him. Then the boy asks how much the disabled dog costs. The owner initially refuses to sell the dog because it has a damaged leg and it will not be the kind of pet the child wants. The child finally buys the dog. As the child is leaving the pet shop the owner realizes that the child is limping and also has a damaged leg.

#### Procedure

The children watched the video in groups of 5–10. Immediately after having seen the video, they were randomly divided into each of the four conditions. The children were then interviewed individually. Children in the EO/EO condition were asked to keep their eyes open throughout the whole interview. Children in the EC/EC condition were asked to keep their eyes closed during the whole interview. In the EO/EC condition children kept their eyes closed only while answering and in the EC/EO condition they closed their eyes only when listening to the questions. If children opened their eyes at times when their eyes should have been closed the children were reminded to close them.

Children were asked a single free recall question at the beginning of the interview (see question 1 in Appendix 2 in Supplementary material), and then 21 specific questions (questions 2–22). Eleven of the specific questions were about visual aspects of the film and 10 were about auditory aspects. Specific questions were defined following ABE (Ministry of Justice, 2011) as questions that included why, what, who, when and how. The specific questions were asked in a different random order for each child.

#### Coding

For the free recall question (question 1) coding was carried out in the same as for questions 1 and 2 in Experiment 1. Details included references to time (e.g., it was daytime), to people (e.g., gender, names, ages) or animals (e.g., dogs, birds), to objects, (e.g., clothes, money), to places (e.g., houses, pet shop, cage) or to actions, (e.g., counting money, playing basketball, feeding animals). There were almost no confabulations in free recall, and therefore these were not analyzed.

Answers to each specific question were coded as correct, incorrect, or as “don't knows.” What constituted a correct answer was agreed prior to the interviews. The maximum possible number of correct answers for specific questions was 21. Two coders coded open-ended and specific questions for one-third of the participants. The proportion of agreement between the two coders was examined via Cohen's kappa, and there was a good agreement for correct details in open-ended questions *k* = 0.818 (*p* < 0.001), for incorrect details in open ended questions *k* = 0.526 (*p* < 001) and for correct answers to specific questions *k* = 0.703 (*p* < 0.001).

### Results

Table 2 summarizes the findings. Free recall (question 1) was analyzed for the number of details provided, and for correct and incorrect details. One child was excluded, because they did not answer the free recall question. A One-Way ANOVA was performed to compare the number of details given by the children in each interview condition (EO/EO, EC/EC, EO/EC, EC/EO). There was no effect for interview [*F*_(3, 148)_ = 0.39, *p* = 0.75, η^2^ = 0.01]. A similar ANOVA showed there was no effect of interview on the number of correct details reported [*F*_(3, 148)_ = 0.37, *p* = 0.69, η^2^ = 0.01]. A third ANOVA found a significant effect of interview condition for incorrect details [*F*_(3, 148)_ = 3.57, *p* = 0.016, η^2^ = 0.07]. *Post-hoc* tests showed that there were more incorrect answers in the EO/EO interviews (*M* = 1.0, *SD* = 1.1) than in the EO/EC condition (*M* = 0.4, *SD* = 0.8) (*p* = 0.046), though we note that there was only a very small mean number of incorrect details (1 or fewer) in any condition (see Table 2).

**Table 2:** **Mean scores for each interview condition in Experiment 2**.

	**Free recall *M* (*SD*)**			**Specific questions *M* (*SD*)**		
	**No. of details**	**Correct details**	**Incorrect details**	**Correct answers**	**Incorrect answers**	**Don't Knows**
Eyes open/eyes open	8.1 (5.0)	6.4 (3.8)	1.0 (1.1)	11.2 (2.5)	5.9 (2.3)	3.8 (2.4)
Eyes closed/eyes closed	7.3 (4.4)	6.8 (4.0)	0.5 (0.8)	11.4 (2.5)	5.9 (2.3)	3.6 (2.8)
Eyes open/eyes closed	7.6 (4.0)	7.2 (3.8)	0.4 (0.8)	11.4 (2.5)	5.5 (2.8)	3.8 (2.1)
Eyes closed/eyes open	8.3 (4.3)	7.4 (3.7)	0.9 (1.0)	10.8 (2.6)	6.4 (2.4)	3.8 (2.2)

Participants were asked 21 specific questions, so the maximum possible score was 21. The mean scores are shown in Table 2. A One-Way ANOVA was conducted to compare the number of correct answers in each interview condition (EO/EO, EC/EC, EO/EC, EC/EO). The different interviews did not have an effect on the number of correct answers [*F*_(3, 148)_ = 0.44, *p* = 0.73, η^2^ = 0.01]. A similar ANOVA was conducted on the number of incorrect answers given. The different interviews had no effect on the number of incorrect answers [*F*_(3, 148)_ = 0.86, *p* = 0.47, η^2^ = 0.02]. A third ANOVA showed that “I don't know” responses were not affected by interview condition [*F*_(3, 148)_ = 0.11, *p* = 0.95, η^2^ = 0.01].

### Discussion

In Experiment 2 the children performed equally in all four conditions, and did so regardless of whether they were being asked for free recall or answering specific questions about the event.

Contrary to our prediction children in the eyes closed condition (EC/EC) did not perform better in free recall than children in the eyes open condition (EO/EO). This finding is in contrast to the results from Experiment 1 and Natali et al. (2012). However, the lack of an eyes closed effect in free recall in Experiment 2 is the same as in Mastroberardino et al. (2012). In free recall children are simply asked to say as much as they can about the event they experienced, so the procedure for a free recall condition is similar in different experiments and comparable results might be expected. But, as yet, there are no consistent findings for the effects of eyes closed on children's free recall of events.

We had predicted that children would be better when they answered specific questions with their eyes closed throughout the interview (in condition EC/EC) than when they answered and kept their eyes open (condition EO/EO), but there was no difference in performance between these conditions. This was in contrast to Experiment 1 and the previous similar studies with children (Mastroberardino et al., 2012; Natali et al., 2012).

We predicted that children might be better at answering questions if they could look at the interviewer while the question was being asked, but closed their eyes while answering the question (in condition EO/EC) than when they had their eyes open all the time. This followed from research on eye gaze (e.g., Doherty-Sneddon and Kent, 1996; Doherty-Sneddon et al., 2001) that has shown that children can benefit from seeing an interviewer while being questioned, but often spontaneously look away from the interviewer while answering questions. However, we did not find that children were better in the EO/EC condition. This finding suggests that closing eyes does not have the same beneficial effect as gaze aversion while responding to questions. This may be because gaze aversion is a typical part of everyday interaction (Doherty-Sneddon, 2004) that requires little effort, but deliberate eye closure may require more effort and be distracting for children.

As expected, children who closed their eyes while listening to questions, but kept their eyes open while answering (EC/EO) did no better than children in the EO/EO condition. There is no reason to suppose that the EC/EO combination of eye closure would benefit children, because children who have their eyes open while answering questions are subject to similar distractions from the environment when responding as children who keep their eyes open all the time.

Experiment 2 did not find any beneficial effect of eyes closure in interviews with children, and did not support the more positive finding from Experiment 1. In the eyes closed condition (EC/EC) of Experiment 2 the children kept their eyes closed continuously, as they did in Experiment 1, but in Experiment 2 keeping eyes closed all the time did not result in the children performing better than children who had their eyes open. The implications of these contrasting results is considered in the general discussion.

## General discussion

If children can benefit from keeping their eyes closed in interviews this is an important finding. One of the most crucial contexts for interviewing children is when children are questioned as part of a police investigation, and in such a context the child might be the only source of evidence (Ministry of Justice, 2011; Kyriakidou, 2012). Therefore any procedure, like keeping eyes closed, that might elicit more evidence or more accurate evidence is important.

Eye closure may be an effective procedure with adults and this has led to researchers suggesting that eye closure can be an effective technique for interviewing adult eyewitnesses (e.g., Glenberg et al., 1998; Wagstaff et al., 2004; Perfect et al., 2008; Vredeveldt and Penrod, 2012; Vredeveldt et al., 2012). However, the evidence that eyes closed procedures benefit children in interviews is equivocal. Children who close their eyes may remember more in free recall (Experiment 1; Natali et al., 2012), but not always (Experiment 2; Mastroberardino et al., 2012). Eyes closed may benefit children when they are answering questions (Mastroberardino et al., 2012; Natali et al., 2012; and visual questions in Experiment 1), but not always (Experiment 2; and auditory questions in Experiment 1). We also note that eye closure has not always been effective in unpublished studies^1^.

Given the current lack of consistent findings in the research with children we suggest caution in implying that eye closure will necessarily benefit children in police interviews. Interviews in experiments are different from forensic interviews. In an experiment the interview questions are carefully crafted and are all clearly relevant to the event being recalled. In actual interviews the questions are spontaneous, they may be ambiguous, there may be a mixture of question formats including specific, forced choice, yes/no and leading questions, and any question can be repeated multiple times in different ways and by more than one interviewer (Krähenbühl et al., 2010; Kyriakidou, 2012). In this complex context it might be the case that an eyes closed procedure would help a child cope with the difficulty of an actual interview, but this has yet to be demonstrated. A major difference between an interview in an experiment and a police interview is the number of questions. In eyes closed studies participants are asked less than 30 questions, but in a police interview children are asked an average of nearly 200 questions (Krähenbühl et al., 2010). The fact that actual interviews include so many questions means that a police interview takes a long time, well beyond the few minutes that an interview in an experiment takes. It may not be appropriate to ask children in an unfamiliar place (the interview room) with two or more unfamiliar adults (the police interviewers) to keep their eyes closed all the time. In a lengthy interview children could be asked to close their eyes only at certain times, but (as shown in Experiment 2) this may not have beneficial effects. Alternatively, if children do not spontaneously avert their gaze when answering questions in a police interview they could be advised to look away from an interviewer (Doherty-Sneddon and Phelps, 2005). But exactly what procedures would be most effective still needs to be investigated in contexts that are more similar to actual police interviews. As yet, the evidence for eye closure benefiting children's recall of events is mixed, and it may be too early to recommend such a procedure for forensic interviews.

### Conflict of interest statement

The authors declare that the research was conducted in the absence of any commercial or financial relationships that could be construed as a potential conflict of interest.

## Footnotes

^1^We have carried out 10 studies that have included eyes closed conditions with children. These have been as part of undergraduate or postgraduate dissertations. In each study children have been shown an unfamiliar film and/or real life event and the procedure has been the same as in Experiment 1, with children being asked for free recall and/or specific questions about the event. In only one of these other studies have we found a beneficial effect of eye closure. In the other studies there was no difference between children's performance in eyes closed and eyes open conditions. In these 9 studies children performed the same in the eyes closed and eyes open condition; there was no increase in correct responses (and no increase in incorrect responses). Therefore eyes closed did not have a detrimental effect on performance, but nor did it improve the quantity of evidence given by the children. We note these studies as a further caution to advocating eye closure as an effective technique for use with children.
