# Supplementary material for: Inconsistent findings for the eyes closed effect in children: the implications for interviewing child witnesses
Source: Front Psychol. 2014 May 20;5:448. doi: 10.3389/fpsyg.2014.00448 (PMC4064256; doi:10.3389/fpsyg.2014.00448)
Supplement: Supplementary file 1 [file DataSheet1.DOCX]

**Appendix 1. Specific questions in Experiment 1**

**A = auditory question, V = visual question**

1. Because I was not there please can you describe what happened with the girls today? Tell me as much as you know, tell me everything and try not to forget anything.
2. Please give me the best description you can of the person who stole the jacket.
3. What was the name of the girl who demonstrated the magic trick to you? (A. answer: Kim).
4. What was the name of the girl who lost her jacket? (A. answer: Mik).
5. Where did the girls have their dinner in the video? (V. answer: Kim’s house).
6. What kind of food was on the table in the video? (V. answer: pizza).
7. What was Kim holding in order to demonstrate the magic trick? (V. answer: tissues).
8. What colour was Mik’s dress in the video? (V. answer: yellow).
9. What colour was Kim’s t-shirt when she was showing you the magic trick? (V. answer: pink).
10. In the video, how did Mik lose her jacket? (V. answer: it was stolen).
11. What was Mik wearing when she came to the classroom to talk with Kim? (V. answer: glasses, hat, top and short trousers).
12. In the video, what did the thief eat from the table? (V. answer: nothing).
13. What was Kim drinking while showing you the magic trick? (V. answer: nothing).
14. Where was Mik’s jacket in the video? (V. answer: on the stand).
15. What colour was Kim’s hat? (V. answer: grey with pink lines).
16. What colour was the vase on the table in the video? (V. answer: white).
17. What was the song about in the video? (A. answer: a song from a famous Cypriot comedy series).
18. What flavour of ice cream did the girls want in the video? (A. answer: strawberry).
19. Where did Mik say she was going after the dinner in the video? (A. answer: to her grandmother).
20. Where did Mik say she was going before she left the classroom? (A. answer: to the police).
21. Why was Mik late for dinner in the video? (A. answer: the taxi driver was late).
22. What did Mik say to Kim when she came into the classroom? (A. answer: ‘I was looking for you everywhere, I’m stressed’).
23. What did the guy say when he entered the house in the video? (A. answer: ‘what a nice house’).
24. Who gave the jacket to Mik? (A. answer: her sister).
25. What were the girls talking about while having their dinner in the video? (A. answer: Mik’s holidays in Hawaii and Kim’s boyfriend).
26. What did Kim say about the classroom where you were? (A. answer: that it would look better if it was painted green with animals).
27. What did the guy say when he was leaving the house in the video? (A. answer: ‘I’m going to sell this’).
28. Why did Kim not use papers to show you the magic trick? (A. answer: because she said you cannot squeeze and cut them easily).

**Appendix 2 Specific questions in Experiment 2**

**A = auditory question, V = visual question**

1. Please tell me everything you saw in the video today.
2. What colour was the t-shirt that the owner of the pet shop was wearing? (V. answer: black).
3. When the children were playing basketball what name were they shouting? (A. answer: Orestis).
4. What was the weather in the video? (V. answer: sunny).
5. How much did the first dog cost? (A. answer: 500 euros).
6. How many children were playing basketball? (V. answer: five).
7. When the owner of the pet shop was talking on the phone to somebody, what was the name of that person? (A. answer: Nick).
8. What was the boy who bought the dog doing in the park? (V. answer: counting his money).
9. How much money did the boy give for the dog? (V. answer: all he had).
10. What animals did you see in the video? (V. answer: dogs).
11. What animals did you hear in the video? (A. answer: birds).
12. Why did the boy not play basketball with the other children? (V. answer: because he had problems with his leg).
13. What did the boy answer to the pet shop owner when he told him that the dog ‘can neither play nor run’? (A. answer: ‘Neither can I’).
14. What colour was the t-shirt of the boy who bought the dog? (V. answer: white).
15. When the children asked the boy, ‘Pal wanna play?’ what did he answer? (A. answer: ‘No’).
16. What colour was the dog the boy bought? (V. answer: white).
17. What did the owner of the pet shop answer when the boy asked him, ‘Can I have a look?’ (A. answer: ‘Go for it’).
18. How many people were wearing glasses? (V. answer: one).
19. What song was playing at the beginning of the video? (A. answer: pink sheep - this was a song from a popular comedy series).
20. How many girls did you see in the video? (V. answer: none).
21. What was the first thing the owner of the pet shop said to the boy when he entered? (A. answer: ‘Hey kid, how can I help you?’).
22. Why did the owner of the pet shop not want to sell the dog to the boy? (A. answer: because there was a problem with the dog’s leg).
